# Workability, quality of life and cardiovascular risk markers in aging nightshift workers: a pilot study

**DOI:** 10.1007/s00508-021-01928-6

**Published:** 2021-09-06

**Authors:** Galateja Jordakieva, Lovro Markovic, Walter Rinner, Isabel Santonja, Seungjune Lee, Alexander Pilger, Thomas Perkman, Igor Grabovac, Eva Schernhammer, Richard Crevenna, Kyriaki Papantoniou, Jasminka Godnic-Cvar

**Affiliations:** 1 Department of Physical Medicine, Rehabilitation and Occupational Medicine, Medical University of Vienna, Vienna, Austria; 2 Institute of Neurology, Medical University of Vienna, Vienna, Austria; 3 Department of Epidemiology, Center for Public Health, Medical University of Vienna, Kinderspitalgasse 15, 1st floor, Vienna, Austria; 4 Center of Virology, Medical University Vienna, Vienna, Austria; 5 Department of Social and Preventive Medicine, Center for Public Health, Medical University of Vienna, Vienna, Austria; 6 Department of Laboratory Medicine, Medical University of Vienna, Vienna, Austria; 7 Channing Division of Network Medicine, Brigham and Women’s Hospital and Harvard, Medical School, Boston, MA USA; 8 Department of Epidemiology, Harvard T.H. Chan School of Public Health, Boston, MA USA

**Keywords:** WAI, WHOQOL, Night shift work, Biomarkers, Aging workers

## Abstract

**Background:**

In aging healthcare professionals, multiple stressors such as night work may affect life and work satisfaction and risk for chronic diseases (e.g. cardiovascular disease [CVD]). In this pilot study we compared workability, quality of life (QoL), and CVD risk markers between night shift and day workers.

**Methods:**

We included 70 hospital employees (mean age 52 ± 4 years, 91.4% female): 32 rotating night shift workers (> 3 nights/month) and 38 permanent day workers. In addition to sociodemographic, lifestyle, and sleep characteristics, we assessed i) workability index (WAI), ii) QoL (World Health Organization Quality of Life [WHOQOL-Bref]) and iii) CVD risk markers, i.e. carotid ultrasound measurements, and biomarkers (NTproBNP, CRP, IL‑6, LDL, ferritin, copper, zinc, and selenium). WAI, QoL, and CVD risk markers were compared between night and day workers. In a subgroup of participants (*N* = 38) with complete data, we used quantile regression analysis to estimate age and multivariate adjusted differences in biomarker levels.

**Results:**

We found no differences in the domains of QoL (physical health, psychological, social relationships, and environment) and WAI scores between night and day workers. Night shift workers were less likely to report excellent workability than day workers, although differences were not statistically significant. Night shift workers reported more sleep problems (73.1% vs. 55.6%) and tended to have lower zinc levels and higher inflammatory markers (CRP, IL‑6, ferritin), but differences were not significant after adjusting for potential confounders.

**Conclusions:**

Workability, QoL and CVD markers did not significantly differ between rotating night shift and day workers in this small pilot study. Sleep problems and inflammatory marker levels carry implications for occupational health.

**Supplementary Information:**

The online version of this article (10.1007/s00508-021-01928-6) contains supplementary material, which is available to authorized users.

## Background

Occupational health services play a key role in managing healthy aging and workplace factors [1, 2]. With respect to the health challenges of an aging workforce, not only older (> 55 years) but also aging (> 45 years) workers have to be addressed in the promotion of workability and health [3, 4]. Research on work-related risk factors should encompass findings from aging worker cohorts to implement systematic health promotion programs and policies in the workplace early on.

Approximately 17% of employees in Europe work in night and shift work outside the standard working hours. Night workers are employees who are likely to work a certain proportion of their annual work time during the night (Directive 2003/88/EC). Shift workers, both with and without night shifts [5], report lower work-life-balance satisfaction than day workers [6]. On the one hand, working atypical hours can be limiting to social life, e.g., working evening shifts significantly contributed to work-life conflict in night shift workers compared to day workers of the Finnish public sector (*n* = 8931) [5]. On the other hand, specific work schedule characteristics associated with atypical working hours might contribute to perceived job strain. A large cross-sectional survey in Sweden found that specific work schedule characteristics (e.g., < 11 h off between shifts and split duty) have more impact on work-life dissatisfaction than night work or night shifts per se [7]. Besides favorable working conditions, perceived physical and mental health promotes workability and work satisfaction. Accordingly, the few available studies showed significant associations between self-reported quality of life (QoL) and workability in nightshift workers [8, 9]. Working asocial hours not only affects psychological well-being [10] but also increases the risk for several chronic health conditions due to the resulting misalignment of the endogenous circadian system and the sleep-wake cycle [11, 12]. Assessment of early and late indicators of biological and functional aging and oxidative stress responses (e.g. leukocytes telomere length) in hospital workers, showed an association of workability scores with the number of night shifts, age, and the number of (chronic) diseases. The authors further concluded that night-shift frequency (nights/month) is associated with oxidative stress, which might induce premature aging [13] and health decline. Shift workers were reported to have several risk factors specifically contributing to cardiovascular disease (CVD) compared to day workers, such as a higher BMI, dyslipidemia [14] and inflammatory markers associated with CVD risk [14–17], with implications of a dose-response [18] risk increase for CVD and CVD mortality [12, 19].

Healthcare professionals (HCP) in emergency and hospital settings constitute a large group of European night shift workers. Besides ergonomic issues, exposure to biological and physical hazards, long working hours, and shift work contribute to work stress in HCP [20]. As the QoL, perceived health, and workability are associated with health outcomes and mortality [21, 22] in aging workforces, we aimed to assess these three aspects in relation to night shift work. In addition, we evaluated CVD markers, such as carotid ultrasound measurements, body composition analysis, and biomarkers for CVD risk in rotating night shift compared to day workers. In this pilot study, we tested the feasibility of all the procedures for a more extensive planned study on the topic, and we report preliminary results.

## Material and methods

### Participants

Study participants were recruited at the General Vienna Hospital between April 2017 and December 2018 among voluntary participants of health-promoting activities organized by the occupational health service and passed down to hospital employees by the head nurses of 16 clinical departments. Night-workers were defined as employees working a rotating shift system with > 3 night shifts/month and controls were defined as employees on permanent day schedules (day workers). The study was approved by the ethics committee of the Medical University of Vienna and conducted in accordance with the Helsinki Declaration and the principles of good clinical practice (EK Number 1260/2017). Informed consent was obtained from all participants before participation in the study. Participants were eligible if they were full-time workers (> 35 h/week) in the hospital (nurses, nurse assistants, patient transporters, administrative staff) and were 45 years of age or older. Participants were excluded if they had a history of cancer, severe cardiometabolic conditions (e.g., myocardial infarction, angina pectoris, type II diabetes, transient ischemia, stroke), and severe psychological conditions (e.g., major depression).

### Study design

This is a pilot cross-sectional study consisting of questionnaires, anthropometric evaluations, and blood sampling. Participants received a screening questionnaire to determine the final eligibility after consenting to study participation. While the study participants filled out the paper-based questionnaires at their own discretion, blood sampling and anthropometric measurements were conducted primarily on-site by one of the study investigators or the nursing staff themselves. A trained neurologist carried out carotid ultrasound.

#### Study questionnaires

The general study questionnaire collected information on various social and demographic aspects (e.g., employment status, education level, marital status) and lifestyle-related questions (e.g., smoking status, physical activity, smoking habits, caffeine and alcohol consumption) as well as personal medical history, medications, sleep, shift work history (work unit, duration of night shift work, nights worked/month) and a question on self-reported chronotype. In addition, diurnal preference was assessed using the morningness-eveningness questionnaire (MEQ) and the total MEQ score was calculated [23]. Leisure time physical activity was assessed by collecting information on type of activity, intensity (low, medium, high) and duration (hours/week) for each of the reported activities. The sum of weekly hours spent doing any type of physical activity was computed. To assess workability, the workability index (WAI) was used, an occupational health instrument for assessing several dimensions of workability, including current workability in relation to job demands and lifetime best workability level, comorbidities count and estimated impairment arising from disease or limiting conditions, amount of sick leave and subjective workability prognosis [24]. The validated German version of the short form (WHOQOL-Bref) of the WHOQOL-100 was used to assess QoL [25]. Items are divided into five domains, i.e., physical, psychological, level of independence, social relationships, and environment, and scored on a Likert-type scale (ranging from 1 to 5, 1 indicating low or negative perceptions and 5 high or positive ones).

#### Cardiovascular risk markers

Weight and height for BMI calculations and body composition analysis (TANITA Body Composition Analyzer MC-180 MA, Batsch Waagen & EDV, Loosdorf, Austria) including total body fat (% of body weight), basic metabolic rate (BMR, kJ/day), total muscle mass (kg), total bone mass (kg), visceral fat level (range between 1 and 59), total body water (% of body weight) measures were performed by an internal medicine specialist.

Carotid artery intima media thickness (CIMT) measurements were obtained using ultrasound imaging and a standardized protocol [26]. The intima media is represented by the area of tissue starting at the luminal edge of the artery and ending at the boundary between the media and adventitia. The CIMT was measured in plaque-free regions on the posterior (far) wall of the left carotid artery. The far and near walls of the left common artery and carotid bulb area were scanned for the presence of atherosclerotic plaques, defined as a distinct area of the vessel wall protruding into the lumen > 50% of the adjacent intima media layer. Mean and maximum intima media thickness, as well as presence of a carotid plaque, are the outcomes analyzed.

During morning blood sampling, serum was collected for biomarker analysis of inflammatory markers: C‑reactive protein (CRP) and interleukin‑6 (IL-6); CVD risk screening markers: low-density lipoprotein (LDL), high-density lipoprotein (HDL), triglycerides, ferritin, total cholesterol, total cholesterol/HDL ratio and n-terminal pro-brain natriuretic peptide (NT proBNP). All clinical chemical analyses were performed on a cobas® 8000 analyzer (Roche Diagnostics, Rotkreuz, Switzerland) in an accredited central laboratory facility. Finally, trace elements relevant for CVD [27–29] (serum levels of copper, zinc and selenium) were measured by graphite furnace atomic absorption spectrometry. For this, EDTA-serum samples were stored at −20 °C until analyses.

### Statistical analysis

Members of the two groups (night workers versus day workers) were descriptively compared with respect to the main outcomes (WAI, QoL, CVD risk markers) and other sociodemographic, lifestyle, sleep, and work-related characteristics. The arithmetic mean and standard deviation were calculated for scale variables and the absolute and relative frequency (presented as percentage) for categorical variables. In univariate analysis, either the *t*-test, Wilcoxon-Mann-Whitney test, χ^2^-test, or Fisher’s exact test were used, depending on the type, distribution, and the number of participants per category of the variable. In a subgroup of participants (*N* = 38) with complete biomarker and confounder information, we used quintile regression analyses to estimate crude, age-adjusted, and multivariate-adjusted (age, smoking status, BMI, physical activity) differences in biomarker levels and 95% CI between rotating night shift workers and day workers. We report quantile regression at the 50th percentile that produces the difference in the median value of the outcome between night shift workers and day workers. We could not run models for selenium due to the small sample size. Analyses were carried out in SPSS v26.0 (IBM Corp. Released 2019. IBM SPSS Statistics for Windows, Version 26.0. Armonk, NY: IBM Corp) and Stata 14.0 (StataCorp. 2015. Stata Statistical Software: Release 14. College Station, TX: StataCorp LP).

## Results

A total of 70 participants were recruited, with 32 (45.7%) currently working in rotating night shifts and 38 in permanent day work (54.3%). Most of the participants belonged to the nursing staff (*n* = 47, 71.2%); the second-largest group was made up of the administrative staff (*n* = 14, 21.2%), as shown in Table 1. The sample was predominantly female (91.4%), White (59.6%), had completed secondary school (46.9%), was married or in a partnership (67.2%,) and was on average 52 years old (SD = 4.0). About one third smoked at the time of the survey (31.7%), the proportion of those who consumed alcohol was slightly higher (38.6%). As far as the work-related characteristics are concerned, the participants worked an average of 39 h/week in the current schedule at the time of the survey, had spent an average of 21 years in the current rotation plan, and had worked an average of 18 years in alternating shifts.

**Table 1:** Sociodemographic and lifestyle characteristics of study sample according to night shift work (*N* = 70)

Variable *in % (n) or arithmetic mean (standard deviation, SD)*	Total	Rotating night shift workers	Day workers	*p*^a^
	100% (*N* = 70)	45.7% (32)	54.3% (38)	
**Sociodemographic characteristics**				
*Job title*				< 0.001
Nurse	71.2% (47)	93.3% (28)	52.8% (19)	
Head nurse	6.1% (4)	3.3% (1)	8.3% (3)	
Nurse assistant	1.5% (1)	3.3% (1)	0.0% (0)	
Administrative personnel	21.2% (14)	0.0% (0)	38.9% (14)	
*Age (years mean, SD)*	52.0 (4.0)	52.0 (4.0)	52.0 (4.0)	0.691
*Gender*				
Female	91.4% (64)	87.5% (28)	94.7% (36)	0.402
Male	8.6% (6)	12.5% (4)	5.3% (2)	
*Education*				0.369
Compulsory schooling (elementary)	1.6% (1)	0.0% (0)	2.6% (1)	
Secondary	46.9% (30)	38.5% (10)	52.6% (20)	
Secondary with university entrance qualification	29.7% (19)	30.8% (8)	28.9% (11)	
Tertiary (university degree or higher)	21.9% (14)	30.8% (8)	15.8% (6)	
*Marital status*				1.000
Single	12.5% (8)	11.5% (3)	13.2% (5)	
Married or in civil union	67.2% (43)	69.2% (18)	65.8% (25)	
Divorced/widowed	20.3% (13)	19.2% (5)	21.1% (8)	
*Domestic situation (nr. of persons in household)*				0.475
1	25.4% (16)	26.9% (7)	24.3% (9)	
2	25.4% (16)	15.4% (4)	32.4% (12)	
3	23.8% (15)	26.9% (7)	21.6% (8)	
4–5	25.4% (16)	30.8% (8)	21.6% (8)	
*Ethnicity*				0.346
White	59.6% (31)	54.5% (12)	63.3% (19)	
Asian	7.7% (4)	13.6% (3)	3.3% (1)	
Black or Middle East	1.9% (1)	4.5% (1)	0.0% (0)	
Other	30.8% (16)	27.3% (6)	33.3% (10)	
*Work hours per week (mean, SD)*	39.09 (10.54)	41.45 (9.36)	34.92 (11.57)	0.074
*Total years in alternating shifts (mean, SD)*	18.21 (11.92)	26.76 (6.78)	8.02 (8.02)	< 0.001
*Years in current rotation plan (mean, SD)*	21 (10)	24 (9)	14 (10)	0.005
*Night shifts per month (mean, SD)*	–	5.8 (3.1)	–	–
**Lifestyle and sleep-related characteristics**				
*Smoking status*				0.847
Smoker	31.7% (20)	30.8% (8)	32.4% (12)	–
No. of cigarettes per week (mean, SD)	345 (233.18)	300 (279.64)	367.50 (219.41)	0.901
Former smoker	22.2% (14)	19.2% (5)	24.3% (9)	–
Never smoked	46.0% (29)	50.0% (13)	43.2% (16)	–
*Current alcohol consumption*				0.685
Yes	38.6% (22)	41.7% (10)	36.4% (12)	–
No. of alcoholic beverages per week (mean, SD)	1.34 (2.30)	1.48 (2.47)	1.24 (2.19)	0.698
No	61.4% (35)	58.3% (14)	63.6% (21)	–
*Caffeine consumption*				0.253
Yes	87.5% (56)	80.8% (21)	92.1% (35)	–
No. of cups of coffee per day (mean, SD)	2.72 (1.62)	2.42 (1.96)	2.93 (1.30)	0.120
No. of cups of caffeinated drinks per day (mean, SD)	3.02 (2.15)	2.85 (2.74)	3.15 (1.62)	0.179
No	12.5% (8)	19.2% (5)	7.9% (3)	–
*Current sleep problem*				0.159
Yes	62.9% (39)	73.1% (19)	55.6% (20)	–
Nights per week (mean, SD)	3.15 (2.01)	2.79 (1.87)	3.50 (2.12)	0.536
No	37.1% (23)	26.9% (7)	44.4% (16)	–
*Wake up in the middle of the night*				0.447
Yes	69.5% (41)	75.0% (18)	65.7% (23)	
No	30.5% (18)	25.0% (6)	34.3% (12)	
*Trouble falling asleep*				0.001
Yes	55.4% (31)	82.6% (19)	36.4% (12)	
No	44.6% (25)	17.4% (4)	63.6% (21)	
*Daytime functioning impairment due to sleep problem*				0.351
None	13.1% (8)	11.5% (3)	14.3% (5)	
Minimal	31.1% (19)	23.1% (6)	37.1% (13)	
Moderate	37.7% (23)	46.2% (12)	31.4% (11)	
Serious	16.4% (10)	19.2% (5)	14.3% (5)	
Severe	1.6% (1)	0.0% (0)	2.9% (1)	
*Physical activity (hours per week)*	4.39 (2.82)	3.77 (1.86)	4.79 (3.28)	0.568
**Chronotype**				
*Total MEQ score (mean, SD)*^*a*^	58 (9)	57 (9)	60 (10)	0.538
Self-assessment				0.895
Clearly evening type	6.7% (2)	6.7% (1)	6.7% (1)	
Rather evening type	33.3% (10)	26.7% (4)	40.0% (6)	
Rather morning type	23.3% (7)	26.7% (4)	20.0% (3)	
Clearly morning type	36.7% (11)	40.0% (6)	33.3% (5)	

^a^The total morningness-eveningness questionnaire (MEQ) score was calculated by summing the scores of all 19 questions included in the questionnaire

Rotating night shift workers were more frequently nurses (93.3% vs. 52.8%) and had on average longer work hours (41.5 vs. 34.9 h/week) compared to day workers (Table 1). Night workers had accumulated on average 27 years of night work, while day workers also reported an average of 8 years of night shift work during their occupational life. Rotating night shift workers were less likely smokers and caffeine drinkers and reported more frequent alcohol consumption and lower levels of physical activity; however, none of these differences reached statistical significance. Night workers more frequently reported sleep problems (73% vs. 56%), especially trouble falling asleep, and were more likely to report moderate to severe impairment of their daytime functioning due to the sleep problems compared to day workers. We found no chronotype differences between the two groups, using the Horne-Ostberg questionnaire-based score [2] and self-reported chronotype.

Most of the participants rated their health and QoL (*n* = 36, 56.3%) as “good” (Table 2). The WAI, which records the subjective ability to work, had an average score of 47 (SA = 4, on a scale of 7–49 points), which corresponds to the category very good. We found no differences in the main domains of QoL (physical health, psychological, social relationships, and environment) and WAI total score between rotating night and day workers. Night workers were more likely to be very satisfied with their capacity for work (38.5% vs. 29.7%), more likely to be satisfied or very satisfied with their own health (80.8% vs. 68.4%), but less likely to rate their own QoL as very good (15.4% vs 23.7%), compared to day workers. Night workers were less likely to have an excellent workability score according to the WAI index, compared to day workers (63.6% vs. 87.5%); however, none of the reported differences in QoL and WAI were statistically significant. Concerning other health-related data, the study participants were predominantly overweight with a mean BMI of 25.7 (SA = 5.0) (Table 3). We found no difference in BMI, body composition analysis, and carotid intima media thickness ultrasound measurements between rotating night and day workers. Zinc levels were significantly lower in rotating shift workers compared to day workers (*p* = 0.046). Mean levels of NT proBNP, CRP, and IL‑6 were not significantly higher in rotating night workers than day workers. In age-adjusted quintile regression models, current night workers had median IL‑6 levels 1.02 pg/ml higher, median NT proBNP levels −18.5 pg/ml lower, and median cholesterol levels −26.5 mg/dl lower than day workers (*p* < 0.05) (Table 4); however, in multivariate-adjusted analyses, none of these differences remained statistically significant. Some differences were found for other quantiles for a few of the biomarkers examined but results should be interpreted with caution due to the small sample sizes (Supplementary Tables 1 and 2).

**Table 2:** Quality of life and workability index (WAI) of study population according to night shift work

Variable *in % (n) or arithmetic mean (standard deviation, SD)*	Total	Rotating night shift workers	Day workers	p*
**Quality of life (WHO-QoL-BREF)**				
Domain 1 (physical health)	16.59 (1.92)	16.80 (1.85)	16.45 (1.97)	0.462
Domain 2 (psychological)	16.26 (2.03)	16.51 (2.01)	16.08 (2.05)	0.783
Domain 3 (social relationships)	15.41 (2.49)	15.46 (1.92)	15.37 (2.84)	0.972
Domain 4 (environment)	16.11 (2.26)	16.11 (1.82)	16.11 (2.54)	0.661
*Satisfaction with own capacity for work*				0.652
Dissatisfied	1.6% (1)	0.0% (0)	2.7% (1)	
Neither satisfied nor dissatisfied	7.9% (5)	11.5% (3)	5.4% (2)	
Satisfied	57.1% (36)	50.0% (13)	62.2% (23)	
Very satisfied	33.3% (21)	38.5% (10)	29.7% (11)	
*Assessment of own quality of life*				0.958
Very poor	0.0% (0)	0.0% (0)	0.0% (0)	
Poor	4.7% (3)	3.8% (1)	5.3% (2)	
Neither poor nor good	18.8% (12)	15.4% (4)	21.1% (8)	
Good	56.3% (36)	65.4% (17)	50.0% (19)	
Very good	20.3% (13)	15.4% (4)	23.7% (9)	
*Satisfaction with own health*				0.569
Dissatisfied	9.4% (6)	7.7% (2)	10.5% (4)	
Neither satisfied nor dissatisfied	17.2% (11)	11.5% (3)	21.1% (8)	
Satisfied	56.3% (36)	65.4% (17)	50.0% (19)	
Very satisfied	17.2% (11)	15.4% (4)	18.4% (7)	
**Work ability index (WAI)**				
*WAI total score (mean, SD)*	47 (4)	47 (5)	47 (4)	0.941
*Work ability category*				0.134
Excellent	77.8% (21)	63.6% (7)	87.5% (14)	
Good	18.5% (5)	27.3% (3)	12.5% (2)	
Moderate	3.7% (1)	9.1% (1)	0.0% (0)	
Poor	0.0% (0)	0.0% (0)	0.0% (0)

**Table 3:** Cardiovascular disease (CVD) risk markers and anthropometric measures of study sample according to night shift work

Variable *in % (n) or arithmetic mean (standard deviation, SD)*	Total	Rotating shift workers	Day workers	p*
*Anthropometric measures*				
Body mass index, BMI (mean, SD)	25.66 (4.98)	25.89 (6.77)	25.53 (3.84)	0.528
Total body fat (% of body weight) (mean, SD)	30.22 (6.58)	31.25 (6.53)	29.68 (6.65)	0.585
Basic metabolic rate, BMR (kJ/day) (mean, SD)	1420 (204)	1410 (244)	1425 (183)	0.389
Total muscle mass (kg) (mean, SD)	45.44 (6.33)	45.07 (7.34)	45.62 (5.87)	0.533
Total bone mass (kg) (mean, SD)	2.43 (0.33)	2.40 (0.39)	2.44 (0.31)	0.469
Visceral fat level (Range 1 to 59)	6 (2)	6 (3)	6 (2)	0.840
Extracellular water (% of body water) (mean, SD)	43.62 (2.87)	44.50 (1.57)	43.15 (3.30)	0.145
Total body water (% of body weight) (mean, SD)	49.61 (4.84)	48.75 (4.74)	50.04 (4.90)	0.577
*Carotid intima media thickness (CIMT)*				
Average CIMT of left ACC/ACI/ACE	0.598 (0.088)	0.589 (0.077)	0.603 (0.095)	0.788
Maximum CIMT of left ACC/ACI/ACE	0.72 (0.11)	0.70 (0.09)	0.73 (0.12)	0.656
Average CIMT of right ACC/ACI/ACE	0.57 (0.10)	0.57 (0.09)	0.57 (0.11)	0.890
Maximum CIMT of right ACC/ACI/ACE	0.69 (0.11)	0.68 (0.09)	0.70 (0.12)	0.682
*Blood CVD biomarkers*				
C‑reactive protein (CRP) (mg/dl)	0.19 (0.24)	0.25 (0.31)	0.15 (0.17)	0.061
Interleukin‑6 (IL‑6; pg/mL)	2.26 (1.35)	2.49 (1.68)	2.13 (1.13)	0.590
Ferritin (mg/dl)	77.30 (62.06)	79.07 (65.22)	76.29 (61.13)	0.965
NT pro Brain natriuretic peptide (NT proBNP) (pg/ml)	62.13 (48.42)	66.78 (54.27)	59.47 (45.37)	0.773
Triglycerides (mg/dl)	117 (56)	117 (50)	118 (60)	0.779
High-density lipoprotein (HDL; mg/dl)	66 (18)	65 (19)	67 (17)	0.739
Low-density lipoprotein (LDL; mg/dl)	115.63 (28.21)	106.48 (21.18)	120.86 (30.59)	0.128
Cholesterol (total; mg/dl)	205 (31)	195 (26)	211 (33)	0.104
Cholesterol (total)/HDL ratio	3.29 (0.87)	3.17 (0.71)	3.36 (0.95)	0.887
Selenium (µg/l)	84.75 (11.26)	89.10 (16.97)	80.40 (4.10)	1.000
Zinc (mg/l)	0.92 (0.18)	0.85 (0.18)	0.96 (0.17)	0.046
Copper (mg/l)	1.15 (0.29)	1.14 (0.22)	1.15 (0.32)	0.624

**Table 4:** Differences in median (Q50) cardiovascular disease biomarker levels and 95% CI confidence intervals (CI) between current rotating night shift workers (*N* = 17) and day workers (*N* = 21) applying quantile regression

	Crude difference (95% CI)	Age-adjusted difference (95% CI)	MV-adjusted difference (95% CI)^a^
C‑reactive protein (CRP) (mg/dl)	0.02 (−0.08, 0.12)	0.03 (−0.10, 0.17)	0.01 (−0.22, 0.23)
Interleukin‑6 (IL‑6; pg/mL)	1.00 (−0.11, 2.11)	1.02 (0.03, 2.01)	0.67 (−0.6, 1.94)
Ferritin (ng/mL)	−15.5 (−67.8, 36.9)	−0.9 (−29.5, 27.7)	−2.95 (−52.2, 46.3)
Brain natriuretic peptide (pg/mL)	−17.2 (−34.2, −0.19)	−18.5 (−1.8, −35.2)	−14.8 (−40.9, 11.3)
Triglycerides (mg/dL)	−23.8 (−65.0, 17.5)	−15 (−67, 37)	−31.0 (−73.6, 11.7)
High-density lipoprotein (HDL; mg/dl)	−8.0 (−26.1, 10.1)	−6.8 (−24, 10.5)	−2.61 (−21.2, 15.9)
Low-density lipoprotein (LDL; mg/dl)	−2.7 (−25.9, 20.5)	−13.2 (−42.5, 16.2)	−15.9 (−54.9, 23.0)
Cholesterol (total; mg/dl)	−24.5 (−43.8, −5.2)	−26.5 (−15.1, −37.9)	−18.9 (−54.1, 16.3)
Cholesterol (total)/HDL ratio	0.2 (−0.51, 0.91)	−18.5 (−37.1, 0.18)	−0.34 (−1.18, 0.50)
Zinc (mg/l)	−0.06 (−0.17, 0.05)	−0.11 (−0.24, 0.02)	−0.15 (−0.37, 0.06)
Copper (mg/l)	0.01 (−0.22, 0.24)	−0.04 (−0.22, 0.15)	0.05 (−0.30, 0.39)

^a^adjusted for age, smoking status (never, former, current), BMI (continuous), hours/week of physical activity (continuous)

## Discussion

In the present pilot study, we assessed workability, QoL, and CVD risk markers in a sample of aging predominantly female rotating night shift and permanent day working hospital employees. We tested the feasibility of study procedures and conducted preliminary analyses of all measured parameters. We found no differences in QoL and workability between rotating night shift and day workers. We found minor differences in CVD biomarkers (lower zinc levels and higher CRP, IL-6) that need to be confirmed in a larger sample.

### Night shift work and workability

While more day workers than night shift workers rated their workability as excellent, WAI scores showed no statistically significant differences between groups. Literature on the association of night shift work with WAI scores is inconclusive. Data from a large Croatian study in hospital employees (*N* = 1856), conducted by Mustajbegovic et al., indicated that despite excellent overall reports of workability, lower WAI scores correlated with shift work, age, and female sex [30]. In an Iranian study including health professionals and administrative employees, WAI scores were associated with BMI, sleep quality, work schedule, work-related stress, and work-life balance [31, 32]. Data from the European Nurses’ Early Exit Study (*N* = 7516) showed that sleep quality and psychosocial factors positively impacted workability levels, regardless of work schedule. While sleep quality and quantity did not seem to mediate the effects of work schedule on workability, the authors reported that sleep and working time satisfaction gradually declined with an increase in proportions of night compared to day work. On the other hand, work involvement, motivation, and financial satisfaction were highest in permanent night workers [33]. Perhaps due to similar factors, working night shifts was associated with higher workability scores in a Portuguese cohort [8]. Besides a link between low workability scores and stressful working conditions, lower WAI scores were associated with increased BMI, sleep problems, and fatigue in nurses (age 35 ± 10 years) at a Brazilian public hospital study [34]. A Croatian study reported lower WAI scores, in correlation with professional demands and outcomes of the physical WHOQOL-Bref domain (odds ratio, OR = 0.78; *p* < 0.001), which encompasses the evaluations of self-reported fatigue, sleep and rest, in shift working emergency medicine staff [9]. In our study participants, a higher prevalence of self-reported sleep problems observed among night workers had no significant impact on total workability scores.

### Night shift and quality of life (QoL) and sleep

Overall, we found no statistically significant differences in the four QoL domains (physical health, psychological, social relationships, and environment) between rotating night shift and day workers. Most of our participants scored high on all domains of the WHOQOL-Bref, despite many years of rotating night shift work. Workability satisfaction and self-assessed QoL were rated good or excellent by the majority of the study participants. Working night shifts was previously associated with negative outcomes in the psychological and environmental domains of the WHOQOL-Bref in white-collar jobs [35]. Fatigue, which can result from disrupted sleep in night shift workers, appears to be a crucial disruptor of QoL, affecting lifestyle and physical activity [8]. Shift work disorder, i.e., reported insomnia or excessive sleepiness (> 1 month) subjectively relevant to shift work schedules, was not only commonly reported (24.4%) but also related to QoL and severe depressive symptoms in Japanese nurses (*N* = 1202). The authors showed that night working hours, nap frequency during night shifts, and chronotype were associated with excessive sleepiness, negatively impacting health-related QoL [36]. Although in our study, sleep disorders were more frequently reported by rotating night shift workers compared to day workers, we found no significant reduction in their QoL; however, it should be noted that in our study sample, day workers were also affected by sleep problems, possibly due to their past exposure to night work, which may lead to long-lasting sleep complaints that persist after workers quit night shift work, as suggested by a recent survey among Austrian hospital employees and the general population [37]. This study also showed that different shift schedules might affect, to a different extend, the risk of sleep problems and subsequent impaired daytime functioning. Interestingly, out of several work time organization schedules, French critical care nursing and paramedical employees working 10‑h night shifts had the lowest QoL scores. The authors found that while specific work time scheduling could influence the physical QoL domain, neither fatigue outcomes nor the mental QoL domain were significantly affected [38]. In our study, rotating night workers worked on average 6 night shifts/month, which is a non-intense rotating night shift schedule that might be tolerated well by aging workers and may not lead to higher levels of fatigue and significant changes in QoL. On the other hand, permanent day workers often work on regular early morning schedules, which may result in sleep problems and fatigue among workers with a late chronotype.

### Night shift and cardiovascular disease (CVD) risk biomarkers

This pilot study found no differences in ultrasound CIMT measurements between night and day workers. Most CVD biomarkers did not differ between the two groups, except for lower zinc and higher IL‑6 and CRP levels among night workers. Night shift work is associated with increased CVD risk [12, 18, 19] and several risk factors potentially contributing to CVD and increased risk for CVD [14]. Measurement of subclinical markers of CVD risk such as CIMT have only sporadically been considered in relation to night shift work, and results are ambiguous. One study among subjects > 45 years found an association between shift work and atherosclerosis, showing an increased CIMT [39]. A more recent study showed that night shift work significantly decreased vascular functions, such as arterial stiffness, especially after long-term shift work but did not affect intima media thickness [40]. Our study results indicated slightly higher inflammatory markers in night shift workers (IL‑6, CRP) than day workers but results were not statistically significant. Our findings are in line with most of the current knowledge on the inflammatory effects of shift work originating in experimental studies showing that sleep deprivation may contribute to higher levels of inflammatory markers, such as IL‑6, CRP, and TNF-alpha [41]. Only a few observational studies have tested this hypothesis among shift workers in real-life settings. An increase in inflammatory markers, such as IL‑6 and CRP [14, 15], and leukocyte counts [16, 17] have been associated with working night shifts. In a prospective study among 8000 British doctors, both night shift work and early morning work were associated with risk factors (CRP, BMI, total cholesterol, triglycerides, HbA1c) for CVD [42]. Shift workers had a higher BMI, CRP, total cholesterol, triglycerides, and LDL cholesterol, compared to day work controls in one field study among cocoa-processing workers [14]. Particularly permanent night shift work has been associated with significant dyslipidemia compared to rotating shift work [43]; however, we observed no differences in LDL, HDL, triglycerides, and no difference in BMI between night and day workers. Zinc levels were significantly higher in the day workers compared to the rotating night shift workers in the entire sample, although in the smaller sample with complete confounder association, this association was not significant. Zinc is protective against acute redox stress in cardiomyocytes and prevents inflammatory processes triggered during myocardial damage [28], thus conveying protective effects in CVD and cardiomyopathy. Little is known about the effects of night shift work on zinc levels; however, zinc supplementation was previously associated with improved sleep quality in ICU nurses [44]. The association between night shift work and zinc levels is interesting and needs to be confirmed in larger studies and in confounder-adjusted analyses. No other CVD biomarker differed significantly between the groups. Several reasons could explain this. First, since this was a pilot study, the sample size was not large enough to detect significant differences, if any existed. Second, our study sample appears to have consisted of predominantly healthy participants for their age. This was also indicated by the very low percentage of reported chronic diseases in both groups. Thus, healthy worker effect and in particular healthy night shift worker effect, participants who tolerate night shift work better are selected into night shift work and remain for longer periods, might explain the lack of associations between night shift work and CVD risk markers. Alternatively, a non-intense rotating night shift work schedule (average of 5 nights/month) might not be as detrimental to cardiovascular health as more intense rotating night shift work schedules and permanent night shifts. While the association between night shift work and low to moderated cardiovascular risk seems established in epidemiological studies, the exact mechanisms and the impact of different shift work schedules on CVD risk factors remain unknown. In the larger project, we aim to evaluate if rotating night shift work of low intensity impacts CVD risk factors in hospital workers.

### Implications for prevention

In terms of prevention, atypical working hours (e.g., shift, night, or weekend work) appear not necessarily negatively related to workers’ well-being, especially with high job control and when working time arrangements are in favor of work–life balance [45]; however, individual assessment of night shift work sensitivity and changes in sensitivity with age and health status, as well as stress management training might be a helpful approach [46], with adaptation of work schedules, particularly in sensitive individuals. Fatigue among shift workers with sleep problems might be reduced by decreasing the proportion of night shifts and giving preference to quickly forward-rotating shift systems [47]. Additionally, recommendations on health-promoting measures aimed at obesity, caffeine consumption, smoking, and sleep disorders appear relevant in the aging workers of our study. Regular health examinations encompassing cardiovascular risk factors should be targeted mainly at night shift workers, who in our study were mostly nursing staff and, thus, have a generally better understanding of health and disease but may pay too little attention to their own health—a generation trait in older Austrians and health workers in general.

### Study feasibility

With emerging evidence on the role of night shift work in QoL, workability and higher risk for CVDs, this pilot study was conducted to examine the feasibility of conducting a larger molecular epidemiological study on this topic. This pilot supports the feasibility and initial acceptability of the proposed procedures among aging hospital employees in the suggested hospital setting. Participants overall responded positively to the study invitation, 70 were recruited, filled out questionnaires, and came in for body analysis, carotid ultrasound, and biological sampling collection. Most procedures (questionnaires, body composition analysis, biological sample collection, and biomarker analysis) are feasible and gave high-quality data. Biomarker and CIMT analyses were less well powered due to study participants not attending blood sampling or ultrasound. Our results require validation in a larger population. The study team is considering offering incentives and shortening the questionnaire length in order to increase participation rates and improve the chances of participants completing all study procedures in the larger study.

### Strengths and limitations

While the extent of our conducted health examinations is a strength of this study, there are some limitations regarding our results. Firstly, due to the feasibility character of our study, sample size did not allow multivariate statistical analysis and the power was limited to detect significant differences between the groups if they exist. Secondly, since participation in the study was voluntary and conducted without a specific incentive for study adherence, several measurements were affected by missing data, thus limiting our analytical sample. Lastly, and due to the same reason, a selection bias leading to a selection in our study of particularly healthy workers cannot be excluded. In our pilot project, we did not have enough power to evaluate individual risk differences in subgroups. Still, in the larger study, we plan to evaluate susceptibility to night shift work in relation to chronic disease risk in relevant subgroups taking into account shift work adaptation, shift work tolerance and chronotype.

### Conclusions

In our study of aging hospital employees, we found no significant differences in workability and quality of life (QoL) between night and day workers. Our study is to our knowledge the first to describe lower zinc levels, a micronutrient with anti-inflammatory properties previously associated with cardiovascular disease (CVD) and sleep quality, in night shift workers; however, in a reduced sample of our data, this association was not significant after adjusting for potential confounders. The reported higher levels of inflammation markers (C-reactive protein [CRP], interleukin-6 [IL-6]) are in line with previous studies showing a potentially elevated CVD risk in night shift workers, which should be considered in occupational health and disease prevention.

## Supplementary Information

Supplementary Tables 1 and 2
