# Supplementary material for: Workability, quality of life and cardiovascular risk markers in aging nightshift workers: a pilot study
Source: Wien Klin Wochenschr. 2021 Sep 6;134(7-8):276–85. doi: 10.1007/s00508-021-01928-6 (PMC9023427; doi:10.1007/s00508-021-01928-6)
Supplement: Supplementary file 1 — Supplementary Tables 1 and 2 [file 508_2021_1928_MOESM1_ESM.docx]

**Supplemental Materials**

**Supplementary Table 1. Age-adjusted differences in quantile (Q10, Q25, Q50, Q75, Q90) cardiovascular disease biomarker levels between current rotating night shift workers (N=17) and day workers (Reference group; N=21) applying quantile regression.**

|  | **Difference (95%CI) ^[a]^** | | | | |
| --- | --- | --- | --- | --- | --- |
|  | Q_15_ | Q_25_ | Q_50_ | Q_75_ | Q_85_ |
| C-Reactive Protein (CRP) (mg/dl) | 0 (NA) | 0.02 (-0.01, 0.06) | 0.03 (-0.10, 0.17) | 0.07 (-0.30, 0.42) | 0.22 (-0.47, 0.92) |
| Interleukin-6 (IL-6; pg/mL) | 1.03 (-0.08, 2.14) | 1.35 (0.17, 2.53) | 1.02 (0.03, 2.01) | 0.61 (-0.45, 1.65) | 0.96 (-0.87, 2.79) |
| Ferritin (ng/mL) | 2.06 (-27, 32) | -16.6 (-60.1, 26.8) | -0.9 (-29.5, 27.7) | 16.3 (-16.7, 49.3) | 20.7 (-15.8, 57.1) |
| Brain natriuretic Peptide (pg/mL) | 16.3 (-10.7, 43.4) | 6.5 (-23.9, 37.0) | -18.5 (-1.8, -35.2) | -18.2 (-60, 23.7) | -16 (-96.7, 64.7) |
| Triglycerides (mg/dL) | -19 (-42, 5.0) | -22 (53, 8.7) | -15 (-67, 37) | -13 (64, 37) | 4.5 (-92, 1.01) |
| High-Density Lipoprotein (HDL; mg/dl) | 2.9 (-9.4, 15.2) | 6.86 (-9.8, 23.5) | -6.8 (-24, 10.5) | -7 (-24.7, 10.7) | -5.5 (-33.3, 22.3) |
| Low-Density Lipoprotein (LDL; mg/dl) | -27.8 (-3.15, -52.4 ) | -17.8 (-48.0, 12.4) | -13.2 (-42.5, 16.2) | -12 (-36.3, 12.3) | -14.5 (-45.7, 16.7) |
| Cholesterol (total; mg/dl) | -28.6 (-61.6, 4.43) | -29.5 (-63.5, 4.47) | -26.5 (-15.1, -37.9) | -32.4 (-70.7, 5.9) | -47.8 (-1.50, -94.0) |
| Cholesterol (total)/HDL-Ratio | 16.3 (-11.2, 43.8) | 6.54 (-18.8, 31.9) | -18.5 (-37.1, 0.18) | -18 (-87.7, 51.3) | -16 (-167, 135) |
| Zinc (mg/l) | -0.14 (-0.39, 0.11) | -0.14 (-0.33, 0.06) | -0.11 (-0.24, 0.02) | -0.10 (-0.22, 0.03) | -0.08 (-0.22. 0.06) |
| Copper (mg/l) | -0.05 (-0.33, 0.24) | -0.03 (-0.30, 0.24) | -0.04 (-0.21, 0.14) | -0.07 (-0.26, 0.12) | -0.05 (-0.64, 0.54) |
| [a] age-adjusted, | | | | | |

| **Supplementary Table 2. MV-adjusted differences in quantile (Q10, Q25, Q50, Q75, Q90) cardiovascular disease biomarker levels between current rotating night shift workers (N=17) and day workers (Reference group; N=21) applying quantile regression.** | | | | | |
| --- | --- | --- | --- | --- | --- |
|  | **Difference (95%CI) ^[a]^** | | | | |
|  | Q_15_ | Q_25_ | Q_50_ | Q_75_ | Q_85_ |
| C-Reactive Protein (CRP) (mg/dl) | 0.05 (-0.2, 0.3) | 0.05 (-0.09, 0.18) | 0.01 (-0.22, 0.23) | 0.11 (-0.15, 0.38) | 0.28 (0.03, 0.54) |
| Interleukin-6 (IL-6; pg/mL) | 0.88 (0.03, 1.74) | 0.52 (-0.4, 1.44) | 0.67 (-0.6, 1.94) | 1.21 (-0.42, 2.84) | 1.28 (-0.39, 2.94) |
| Ferritin (ng/mL) | -20.1 (-97.7, 57.4) | -6.3 (-70.6, 58.0) | -2.95 (-52.2, 46.3) | 11.3 (-52.7, 75.2) | -1.22 (-68.2, 65.8) |
| Brain natriuretic Peptide (pg/mL) | 13.0 (-31.9, 58.0) | 9.00 (-37.1, 55.1) | -14.8 (-40.9, 11.3) | -9.9 (-53.2, 33-5) | 5.69 (-56.6, 68.0) |
| Triglycerides (mg/dL) | -15.7 (-65.8, 34.5) | -13.8 (-58.0, 40.3) | -31.0 (-73.6, 11.7) | -30 (-71.5, 11.6) | -38.7 (-0.95, 18.6) |
| High-Density Lipoprotein (HDL; mg/dl) | 8.0 (-15.1, 31.0) | 9.6 (-15.9, 35.1) | -2.61 (-21.2, 15.9) | 1.98 (-15.3, 19.3) | 6.83 (-11.1, 24.7) |
| Low-Density Lipoprotein (LDL; mg/dl) | -10.1 (-39.7, 19.5) | -7.65 (-34.7, 19.4) | -15.9 (-54.9, 23.0) | -10.6 (-53.5, 32.3) | -38 (-86.8, 9.83) |
| Cholesterol (total; mg/dl) | -23.9 (-56.9, 9.12) | -22.5 (-53.9, 8.9) | -18.9 (-54.1, 16.3) | -22.0 (-64.2, 20.1) | -28.6 (-75.3, 18.2) |
| Cholesterol (total)/HDL-Ratio | -0.39 (-1.28, 0.49) | -0.37 (-1.23, 0.49) | -0.34 (-1.18, 0.50) | -0.56 (-1.3, 0.21) | -0.64 (-1.56, 0.27) |
| Zinc (mg/l) | -0.14 (-0.54, 0.27) | -0.17 (-0.54, 0.19) | -0.15 (-0.54, 0.24) | -0.09 (-0.41, 0.23) | -0.13 (-0.45, 0.19) |
| Copper (mg/l) | -0.06 (-0.27, 0.16) | -0.09 (-0.31, 0.13) | -0.04 (-0.22, 0.15) | 0.07 (-0.25, 0.38) | 0.01 (-0.35, 0.37) |
| [a] adjusted for age, smoking status (never, former, current), BMI (continuous), hours/week of physical activity (continuous) | | | | | |
